# Preferential Expression of Ca^2+^-Stimulable Adenylyl Cyclase III in the Supraventricular Area, including Arrhythmogenic Pulmonary Vein of the Rat Heart

**DOI:** 10.3390/biom12050724

**Published:** 2022-05-20

**Authors:** Yosuke Okamoto, Naing Ye Aung, Masahiro Tanaka, Yuji Takeda, Daichi Takagi, Wataru Igarashi, Kuniaki Ishii, Mitsunori Yamakawa, Kyoichi Ono

**Affiliations:** 1 Department of Cell Physiology, Akita Graduate School of Medicine, Hondo 010-8543, Japan; 2 Pathological and Image Analysis Center, Cancer Research Center, Faculty of Medicine, Yamagata University, Iida-Nishi 990-9585, Japan; a.naing@med.id.yamagata-u.ac.jp (N.Y.A.); myamakawa236.kamihozawa@gmail.com (M.Y.); 3 Department of Pharmacology, Faculty of Medicine, Yamagata University, Iida-Nishi 990-9585, Japan; breathless.06@icloud.com (M.T.); kuishii@med.id.yamagata-u.ac.jp (K.I.); 4 Department of Immunology, Faculty of Medicine, Yamagata University, Iida-Nishi 990-9585, Japan; yu-takeda@med.id.yamagata-u.ac.jp; 5 Department of Cardiovascular Surgery, Akita Graduate School of Medicine, Hondo 010-8543, Japan; takagi@med.akita-u.ac.jp (D.T.); vvataruig@gmail.com (W.I.)

**Keywords:** adenylyl cyclase, supraventricular area, pulmonary vein, arrhythmia, atrial fibrillation, t-tubule

## Abstract

Ectopic excitability in pulmonary veins (PVs) is the major cause of atrial fibrillation. We previously reported that the inositol trisphosphate receptor in rat PV cardiomyocytes cooperates with the Na^+^-Ca^2+^ exchanger to provoke ectopic automaticity in response to norepinephrine. Here, we focused on adenylyl cyclase (AC) as another effector of norepinephrine stimulation. RT-PCR, immunohistochemistry, and Western blotting revealed that the abundant expression of Ca^2+^-stimulable AC3 was restricted to the supraventricular area, including the PVs. All the other AC isotypes hardly displayed any region-specific expressions. Immunostaining of isolated cardiomyocytes showed an enriched expression of AC3 along the t-tubules in PV myocytes. The cAMP-dependent response of L-type Ca^2+^ currents in the PV and LA cells is strengthened by the 0.1 mM intracellular Ca^2+^ condition, unlike in the ventricular cells. The norepinephrine-induced automaticity of PV cardiomyocytes was reversibly suppressed by 100 µM SQ22536, an adenine-like AC inhibitor. These findings suggest that the specific expression of AC3 along t-tubules may contribute to arrhythmogenic automaticity in rat PV cardiomyocytes.

## 1. Introduction

Ectopic excitability originating in pulmonary veins (PVs) leads to symptomatic atrial fibrillation, the most frequently observed arrhythmia [1]. Several studies have investigated the arrhythmogenic properties of PVs [2,3,4,5,6], and various types of spontaneous activities have been recorded in PV cardiomyocytes, including the sinoatrial node (SAN)-like spontaneous action potentials [2,4], digitalis-induced arrhythmia [7], pacing-induced spontaneous activity after ryanodine treatment [8], and burst-pacing-induced atrial fibrillation after genetic deletion of ryanodine receptor-regulating subunit FKBP12.6 [9]. This arrhythmogenic activity partly depends on the distinct electrophysiological characteristics of cells and involves intracellular Ca^2+^ dynamics [10]. Previous studies have demonstrated the specific mechanisms by which Ca^2+^ contributes to the firing of action potentials under α- and β-adrenoceptor stimulation [3,5,11]. In response to norepinephrine application, local diastolic Ca^2+^ release via the inositol 1,4,5-triphosphate receptor 2 (IP_3_R_2_) activates the Na^+^-Ca^2+^ exchanger (NCX), which accelerates diastolic depolarization. The functional coupling between IP_3_R_2_ and NCX is facilitated by both the structural characteristics of PV cardiomyocytes and the localization of the two molecules, in that PV cardiomyocytes possess enriched t-tubules compared with atrial myocytes, and IP_3_R_2_ co-localizes with NCX in t-tubule microdomains [5,11]. The potential automaticity of PV cardiomyocytes is manifested by the cooperation of spontaneous Ca^2+^ release and an ensemble of sarcolemmal electrogenic molecules, including hyperpolarization-activated K^+^ current (I_KH_) in dog [12] and hyperpolarization-activated Cl^−^ current (I_Cl,h_) in rat [13].

Numerous studies have shown that the cardiac autonomic nervous system is involved in the occurrence and persistence of atrial fibrillation [14]. Indeed, abundant sympathetic nerves innervate human PVs [15]. Norepinephrine released from sympathetic nerve terminals stimulates both α- and β-adrenergic receptors, which in turn activate G_q_- and G_s_-protein-coupled signaling pathways, respectively. The former pathway activates IP_3_R_2_ and thus induces sporadic [Ca^2+^]_i_ elevations [16,17], while the latter pathway involves cyclic-adenosine monophosphate (cAMP) and acts on a variety of downstream effectors, including protein kinase A. Protein kinase A phosphorylation of intracellular targets coordinates a number of physiological outputs, such as myocyte contraction and relaxation and pacemaker activity. The synthesis of cAMP from ATP is controlled by the enzyme adenylyl cyclase (AC). The AC family is composed of nine membrane-bound isoforms (AC1–9) and a soluble form (AC10). Most of the AC isoforms can be found in the heart [18]. While AC5 and AC6 are major isoforms in adult cardiomyocytes [19], AC1 is proposed to function as a Ca^2+^-stimulable AC in the SAN and to contribute to normal pacemaker rhythm [20]. In fact, cAMP activity in SAN cells is enriched compared with that in ventricular cardiomyocytes [21], enabling spontaneous, rhythmic, local diastolic Ca^2+^ releases from storage sites and subsequent activation of the NCX inward current that accelerates diastolic depolarization. It is now believed that the release and uptake of Ca^2+^ via the ryanodine receptor (Ca^2+^ clock) facilitates SAN automaticity with an ensemble of several ion currents on the membrane (membrane clock) [22].

In the present study, we found a unique expression pattern of another Ca^2+^-stimulable AC, AC3, in the supraventricular area and investigated the impacts of its activity on the potential automaticity of PV cardiomyocytes using the patch-clamp technique.

## 2. Materials and Methods

### 2.1. Tissue Preparation

**Figure 1:**
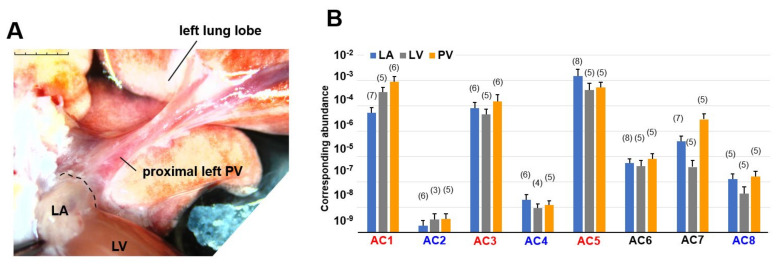
Macroscopic view of the preparation and genetic RT-PCR screening for adenylyl cyclase (AC) superfamily members. (**A**) A stereomicroscopic snapshot of the cardiopulmonary preparation focusing on a left pulmonary vein (PV). To visualize the veins, fresh blood was left inside. The PV extends from the left atrium (LA) and visibly branches into the left superior lung lobe. The post caval lobe of the lung is behind the left PV. The branches from the right PV are out of focus and cannot be fully seen in this view. A part of the left ventricular (LV) mass is observed at the bottom. Scale bar: 5 mm with 1 mm increments. (**B**) Quantitative RT-PCR for AC1–8. By fitting each quantitative cycle to a standard linear curve, the abundance of each reverse-transcribed cDNA is measured quantitatively. The exogenous standard curves are prepared by RT-PCR of diluted RNA extracted from the right atrium. Accordingly, the vertical axis corresponds to the same amount of standard cDNA. The ordinary one-way analysis of variance indicates that expression levels significantly vary from isotype to isotype (*p* < 0.0001). Tukey’s multiple comparisons test identified statistically significant differences. AC1, AC3, and AC5 (in red) are all expressed more strongly than AC2, AC4, and AC8 (in blue). To simplify our PCR data, bar graphs with means ± standard deviations instead of dot plots. The number of experiments is provided in parentheses.

For RNA extraction from different regions of the cardiopulmonary tissues, male Wistar rats (10–15 weeks of age) were euthanized. The rats were deeply anesthetized by intraperitoneal injection of 0.15 mg/kg of medetomidine, 2.0 mg/kg of midazolam, and 2.5 mg/kg of butorphanol. After checking the suppression of the nociceptive reflex, the chest cavity was opened under artificial ventilation, and the aorta was cannulated in situ to perfuse the coronary arteries. The heart and lungs were excised in a block, mounted on a Langendorff apparatus, and sequentially perfused with ice-cold, Ca^2+^-free, and heparinized Tyrode solution for 5 min. The composition of Tyrode solution (in mM) was: NaCl 136.9, KCl 5.4, NaH_2_PO_4_ 0.33, HEPES 5.0, MgCl_2_ 0.5, glucose 5.5; pH 7.4 adjusted with NaOH. The heart-lung block was pinned to a tissue bath. Soft tissue containing the vagus nerve and adipose tissue was trimmed off under a stereomicroscope (Figure 1A). The PV was exposed after excision of the pulmonary trunk; PV samples were collected from the proximal and peripheral left PVs, while right PVs were routinely excluded. The left atrium (LA) adjacent to the PV, the left ventricle (LV) mass, and the right atrium were isolated. Each LV mass was dissected into three pieces (i.e., samples). All cardiopulmonary tissues were isolated within 10 min after removal of the heart from the body. During tissue isolation, the external solution was perfused at a rate of 10 mL/min on ice. All tissue samples were fresh-frozen in liquid nitrogen and stored at −80 °C for later use.

### 2.2. Reverse Transcription-Polymerase Chain Reaction (RT-PCR)

RNA was extracted using RNeasy Mini Columns (Qiagen, Chatsworth, CA, USA) and then reverse-transcribed using the High-Capacity cDNA Reverse Transcription Kit (Applied Biosystems™, Waltham, MA, USA). The transcribed cDNA was amplified by the LightCycler Nano System (Roche Holdings AG, Basel, Switzerland) by monitoring SYBR Green fluorescence. The AC expression levels in the LA, LV, and PV were analyzed by both simplified-absolute quantifications. For absolute quantification with standard linearity, cDNA from the right atrium was diluted 1 to 10^−7^ times in 10× decrements. The dilutions were amplified by the PCR primer for glyceraldehyde-3-phosphate dehydrogenase (GAPDH), and their quantification cycles (Cq) were adopted as an exogenous standard. The accuracies of all the PCR primers designed for the current study (Table 1) were evaluated by a singular peak of the melting curves and the specific bands on the Tris-borate-EDTA gel (1% agar). The specificity of all the primers was confirmed on the NCBI database (https://blast.ncbi.nlm.nih.gov/Blast.cgi (accessed on 1 March 2017). 

### 2.3. Immunohistochemistry

For heart fixation, a similar procedure as for tissue preparation was applied. After the blood was washed out from the heart with Ca^2+^-free Tyrode solution, the heart-lung block was detached from the Langendorff apparatus. To expand the PVs, 10% neutral-buffered formalin solution was injected from the aortic arch and LV free wall into the LV cavity with a 20G needle. The block was immersed in a 10% neutral-buffered formalin solution for 24–48 h. Immunohistochemistry of the rat heart was performed with rabbit polyclonal antibodies against AC1 (Antibodies-Online, Atlanta, GA, USA; cat. no. ABIN702565), AC2 (Abcam, Cambridge, UK; cat. no. ab151470), AC3 (Antibodies-Online; cat. no. ABIN2736239), AC6 (Antibodies-Online; cat. no. ABIN751198), and AC8 (Antibodies-Online; cat. no. ABIN751228) at the manufacturer-recommended concentrations. Next, 3 μm thick sections were cut and deparaffinized. Endogenous peroxidase activity was blocked with methanol containing 0.3% hydrogen peroxide for 30 min on ice. Antigen retrieval was performed using EDTA (Antigen Retrieval Solution pH 9; Nichirei Biosciences, Tokyo, Japan) or citric acid (Antigen Retrieval Solution pH 6; Iatron Laboratories Inc., Tokyo, Japan) in an autoclave (2 atmospheres, 121 °C, 20 min). An EnVision+ System HRP-labeled polymer (anti-rabbit; DAKO, Carpinteria, CA, USA) was used. Positive reactions were detected as brown coloration with 3,3-diaminobenzidine tetrahydrochloride (Dojindo, Kumamoto, Japan). The sections were then counterstained with hematoxylin. The brain cortex was used as a positive control for AC1 and AC3. Lung epithelium cells were used as positive controls for AC2. AC6 was positively stained in the entire heart regions. 

### 2.4. Cell Isolation

To isolate cardiomyocytes from the rat heart, a similar procedure as for tissue preparation was applied. The heart-lung block was mounted on a Langendorff apparatus and sequentially perfused with the following buffers at 37 °C: (1) normal Tyrode solution for approximately 3 min; (2) Ca^2+^-free Tyrode solution for 5 min; and (3) Ca^2+^-free Tyrode solution containing 0.05% collagenase and 0.005% elastase (Wako Pure Chemicals, Osaka, Japan) for 30 min. The composition of the normal Tyrode solution (in mM) was: NaCl 136.9, KCl 5.4, CaCl_2_ 1.8, MgCl_2_ 0.5, NaH_2_PO_4_ 0.33, HEPES 5.0, glucose 5.5; pH 7.4 adjusted with NaOH. After digestion, the heart-lung block was perfused with 50 mL of a high-K^+^ and low-Cl^−^ solution. The composition of the solution (in mM) was: L-glutamic acid 70, KOH 70, KCl 30, KH_2_PO_4_ 10, MgCl_2_ 1, taurine 20, glucose 10, EGTA 0.3, HEPES 10; pH 7.4 adjusted with KOH. The soft tissue containing the vagus nerves, adipose tissue, and PA was trimmed off under a stereomicroscope, and the left PV was excised from the digested block. The PV was minced gently in the high-K^+^ and low-Cl^−^ solution, and the pieces were gently agitated to dissociate the cells. The cell suspension was stored at 4 °C for later use.

### 2.5. Immunocytochemistry

Freshly isolated cardiomyocytes were incubated for 30 min on poly-D-lysine-coated coverslips and subsequently fixed with 4% paraformaldehyde for 30 min at room temperature. The fixed cells were permeabilized with phosphate-buffered saline (PBS) containing 0.2% Triton X-100 for 30 min, blocked with blocking buffer (PBS containing 5% bovine serum albumin), and incubated with primary antibodies against AC3 (Proteintech, Chicago, IL, USA; cat. no. 19492-1-AP) and α-actinin (Abcam; cat. no. ab9465) at 1:500 dilution in PBS containing 1% bovine serum albumin. The cells were then incubated with appropriate secondary antibodies conjugated with either Alexa Fluor 488 or Alexa Fluor 594 (at 1:1000 dilution) and mounted on glass slides with glycerol with 10% 1,4-diazobicyclo-(2,2,2)-octane. Confocal images were obtained using a Zeiss 780 confocal microscope at ×100 magnification (pinhole size of an arbitrary unit). The captured images were processed using ZEN Image software (Zeiss, Oberkochen, Germany). The fluorescence intensity profiles of α-actinin and AC3 for each cell type were analyzed by ImageJ software, and the cross-correlations were calculated in IGOR software ver. 6.36 (Wavemetrics, Portland, OR, USA).

### 2.6. Patch-Clamp Electrophysiology

The conventional and perforated whole-cell patch-clamp configurations were used to record membrane currents with a patch-clamp amplifier (Axopatch 200B; Molecular Devices, Chicago, IL, USA). The composition of normal Tyrode solution (in mM) was: NaCl 136.9, KCl 5.4, NaH_2_PO_4_ 0.33, CaCl_2_ 1.8, HEPES 5.0, MgCl_2_ 0.5, glucose 5.5; pH 7.4 adjusted with NaOH. During the patch-clamp experiments, the normal or Cs-Tyrode were perfused as the external solution. The Cs-Tyrode solution was prepared by replacing KCl with equimolar CsCl for L-type Ca^2+^ recording. The composition of the pipette solution (in mM) for L-type Ca^2+^ recording was: CsOH 120, aspartic acid 80, CsCl 20, Na_2_-ATP 5, MgCl_2_ 5, HEPES 5, Na_2_-GTP 0.1 and EGTA 5 or CaCl_2_ 0.1; pH 7.2 adjusted with aspartic acid. Borosilicate glass electrodes with tip resistances of 2.0–5.0 MΩ, when filled with internal solution, were used. The cell membrane capacitance (Cm) was determined by applying a 30 ms hyperpolarizing voltage-clamp step from a holding potential of −40 mV to −50 mV and integrating the area under the capacitive transient. The Cm of LA, LV, and PV cardiomyocytes were 107.25 ± 20.23 (*n* = 13), 238.41 ± 52.50 (*n* = 9), and 210.91 ± 61.93 (*n* = 28) pF, respectively. L-type Ca^2^+ current was recorded every 10 s, where a cell was held at a potential of −80 mV. The Ca^2+^ current was induced by a depolarizing pulse to 0 mV for 500 ms, followed by a depolarizing pulse to −40 mV for 80 ms to inactivate the Na^+^ current. The series resistance estimated from the Cm value and the time constant of the capacitive transient was 6.05 ± 2.14 MΩ (*n* = 20), selecting 5, 5, and 10 from the LA, LV, and PV cardiomyocyte experiments, respectively. The composition of the pipette solution (in mM) for norepinephrine-induced automaticity was: KOH 110, aspartic acid 110, KCl 30, NaCl 10, HEPES 5, EGTA 10; pH 7.2 adjusted with KOH. Amphotericin B at 0.3 mg/mL was also added. Membrane currents and potentials were recorded under voltage- and current-clamp conditions, respectively, at 37 ± 1 °C. Data acquisition and storage were accomplished with CLAMPEX software ver. 10.7 (Molecular Devices) running on a personal computer. The sampling frequency was 1 kHz, and low-pass filtering was performed at 500 Hz. All patch-clamp data were analyzed using IGOR software ver. 6.36 (Wavemetrics).

### 2.7. Drugs

Chemicals and reagents were purchased from Sigma-Aldrich (St. Louis, MO, USA) or Fujifilm Wako Pure Chemical Corporation. Stock solutions of dl-norepinephrine and dl-isoproterenol were prepared in standard and Cs-Tyrode solution, respectively. Amphotericin B and SQ22536 were dissolved in dimethyl sulfoxide under light-free conditions and diluted 1000-fold for experiments.

### 2.8. Statistics 

Statistical significance was determined by an ordinary one-way analysis of variance (ANOVA) with Tukey’s multiple comparisons test for comparing more than three groups or by the Welch’s *t*-test to compare two groups, using GraphPad Prism ver. 9.0.0. As the higher mean value of the expression levels measured by RT-PCR clearly indicates a higher variance (Figure 1B), we tested the expression levels by a parametric analysis with log transformation. To avoid negative values, a constant (10^9^ + 2) was added to the raw values, logtransformed with a base of two, and then evaluated with a one-way ANOVA. Data were expressed as mean ± standard deviation. Values of *p* < 0.05 (*) and *p* < 0.01 (**) indicated statistical significance.

## 3. Results

### 3.1. Genetic Screening of AC Family Members in Cardiopulmonary Organs by RT-PCR

We performed multiplex PCR analysis to characterize the expression patterns of AC isoforms (AC1–8) in the heart. The activity of AC9 was estimated to be less than 3% of the total AC activity by the previous report [24], and it was not initially considered as a major AC candidate. This is why we did not include AC9 for this screening. AC10 is a soluble AC, not a membrane enzyme. In this study, we focused on membrane enzymes that contribute to arrhythmias and action potentials, and AC10 was not included. In our PCR experiments, total RNAs extracted from three different regions of cardiopulmonary tissues, left atrium (LA), left ventricle (LV), and pulmonary vein (PV) (Figure 1A), were reverse-transcribed and amplified by the LightCycler. We evaluated absolute gene expression levels in each cardiopulmonary region (Figure 1B). In addition to Ca^2+^-inhibitable AC5, known as the major heart isotype [25], Ca^2+^-stimulable AC1 and AC3 were predominantly expressed across the entire heart. AC1 expression was significantly higher than AC2, AC4, AC6, and AC7 expressions, AC3 expression was significantly higher than AC2, AC4, and AC8 expressions, and AC5 expression was significantly higher than AC2, AC4, AC6, AC7, and AC8 expressions (*p* < 0.05, see Figure 1B, and Table 2). 

### 3.2. Immunohistochemistry of AC Family Members in Cardiopulmonary Regions

**Figure 2:**
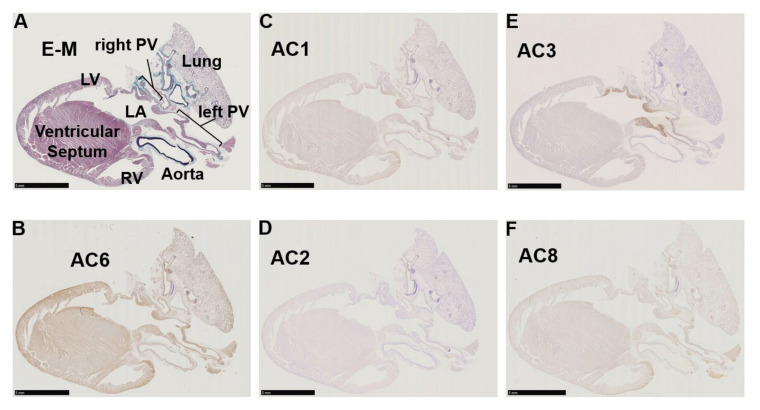
Immunohistochemistry of a left-sided rat heart for adenylyl cyclase AC1, AC2, AC3, AC6, and AC8. (**A**) Masson’s trichrome staining in a left-sided heart from a male Wistar rat. Muscle, collagen-enriched tissue, and elastin-enriched fibers are stained purple, turquoise, and dark blue, respectively. The cardiopulmonary regions are indicated by bold letters. LA, left atrium; LV, left ventricle; PV, pulmonary vein; RV, right ventricle. (**B**) Immunohistochemistry for AC6. AC6 is known as a heart subtype of AC and presents a positive control with solid staining (brown). (**C**–**F**) Immunohistochemistry for AC1–3 and AC8. AC1 and AC8 are both moderately positive in (**C**,**F**). (**D**) AC2 is almost negative in the heart, while the lung epithelium is positively stained. (**E**) AC3 expression is restricted to the LA and PV. Scale bars: 5 mm.

**Figure 3:**
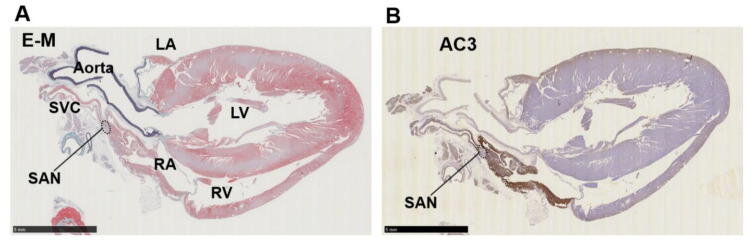
Immunohistochemistry of a right-sided rat heart for adenylyl cyclase AC3. (**A**) Masson’s trichrome staining. LA, left atrium; LV, left ventricle; RA, right atrium; RV, right ventricle, SAN, sinoatrial node; SVC, supra vena cava. (**B**) Immunohistochemistry for AC3. The supraventricular area except the SAN is stained positive. Scale bars: 5 mm.

**Figure 4:**
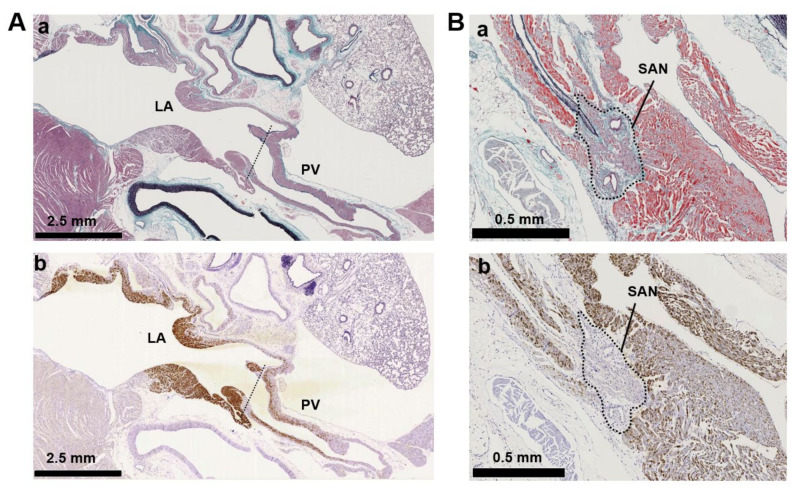
Immunostaining of adenylyl cyclase AC3. (A) Supraventricular regions with Masson’s trichrome staining (Aa) and immunostaining for AC3 (Ab). The ventricular septum is visible beneath the mitral valve on the lower left, a connective tissue-enriched thin structure. The lung is on the upper right. The tentative boundary line between the left atrium (LA) and pulmonary vein (PV) is indicated by a dotted line. (B) Intercaval regions with Masson’s trichrome staining (Ba) and AC3 (Bb) immunostaining. The sinoatrial node (SAN) is defined as a myocardial region surrounded by collagen-enriched connective tissues and contains the SAN artery. The artery is observed as circular cavities. The area indicated by the dotted line is the SAN. The superior vena cava is located immediately above the SAN to the right.

Immunohistochemistry of cardiopulmonary regions was performed to characterize protein expression patterns. To outline the heart structure, modified Masson’s trichrome (M-T) staining of a left-sided heart preparation was performed (Figure 2A). In the M-T-stained preparation, muscular areas (purple) were abundantly observed in not only the LV and LA but also in the left and right PVs. The LV and ventricular septum were separated from the LA by collagen-enriched mitral valves (turquoise blue). The left and right PVs were both identified as tubular structures extending from the LA. All of the structures appeared to consist of striated muscle, consistent with previous reports [26,27]. Immunostaining of AC6 was used as a positive control [28] and showed intense brown staining that was detected throughout the heart and in the PVs (Figure 2B). AC1 was moderately positive (Figure 2C) and distributed throughout the entire heart. AC2, a Ca^2+^-insensitive AC isoform, was almost negative (Figure 2D). Meanwhile, AC3-positive regions were specifically restricted to the LA and PVs. AC8, another Ca^2+^-stimulable AC, was mildly stainable through the entire heart (Figure 2F). The unique expression of AC3 in the supraventricular area was confirmed in the right-sided preparation, except for the sinoatrial node (SAN) (Figure 3B). Classically, SAN is defined as an intercaval heart area surrounded by connective tissues and containing the SAN artery. In the expanded view of Figure 4Ab, a high AC3 expression was uniformly observed in the LA, while the AC3 expression in the PV was enriched on the inner side of the vessel. There was an unstained wall between the visibly stained LA and PV. Peripheral portions of the PV were also unstained. Figure 4B shows the intercaval area in a right-sided rat heart with M-T staining (upper panel) and immunostaining of AC3 (lower panel). The M-T staining identified the SAN as a muscle region containing cross-sections of two arteries, which were surrounded by connective tissues (Figure 4Ba). The corresponding SAN segment was not stained by the anti-AC3 antibody (Figure 4Bb). 

### 3.3. AC3 Is Localized in the t-Tubule Microdomains of PV Cardiomyocytes

**Figure 5:**
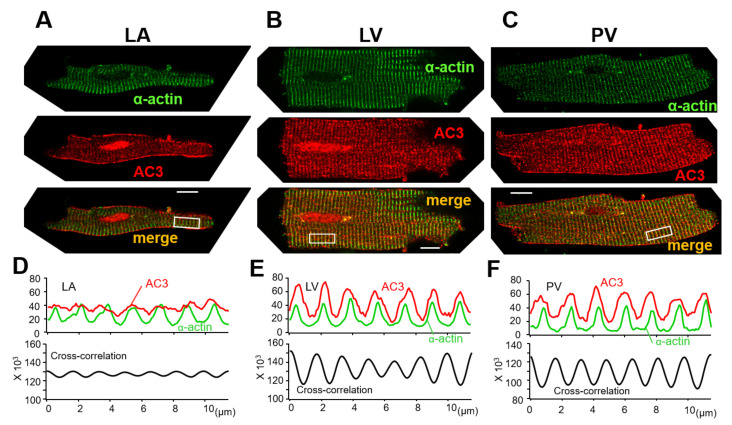
Immunocytochemistry for adenylyl cyclase AC3 in the myocytes of (**A**) isolated left atrium (LA), (**B**) left ventricle (LV), and (**C**) pulmonary vein (PV). α-actinin (green, all top panels) localized to the z-line of cardiomyocytes, indicating the position of the t-tubules. AC3 staining (red, all middle panels) revealed transverse striations in LV and PV myocytes, unlike in LA myocytes. AC3 is strongly expressed along the cell contour in LA myocytes. The bottom panels display the overlay of AC3 on α-actinin (yellow-orange). The white bars in the bottom panel indicate 10 μm. (**D**,**E**) and (**F**) display the profiles of α-actin and AC3 staining intensities in (**A**–**C**), and the cross-correlation between both intensity profiles, respectively. The area to be intensity-profiled is chosen from the rectangular box in the bottom panel of (**A**–**C**), all of the same size. The cross-correlation waveforms indicate that the intensities of actin and AC3 are synchronized, suggesting that they are localized near each other [11,32]. It should be noted that the nuclei of LA and LV are stained with AC3. Neither the laser intensity of the microscope nor the gain during imaging is increased in these imaging compared to PV imaging. Considering that the transverse microstructures can be captured, it is more likely that the nuclei were stained by anti-AC3 antibody rather than overexposure.

Figure 5 shows the cellular locations of α-actinin (green) and AC3 (red) on the freshly isolated LA, LV, and PV cardiomyocytes. α-actinin is localized at the cardiac z-line in each myocyte. LA myocytes display weak transverse striations of AC3, while the AC3 expression along the cell contour was relatively clear (Figure 5A, middle panel). The cross-correlation between α-actinin and the AC3 signal is relatively small (Figure 5D). A previous study revealed that transverse tubules (t-tubules) of the LA were less enriched [5]. Accordingly, blunt striations in AC3 staining and a weak cross-correlation between α-actinin and the AC3 signal are supposed to reflect immature t-tubules of LA cells. Similarly, as with AC5, AC6 [29], and AC9 [30], AC3 was suggested to co-localize with α-actinin in both LV and PV cells (Figure 5B,C,E,F). As the z-line is known to overlap with t-tubules in ventricular myocytes [31], the co-localization of α-actinin and AC3 in these cells indicates that in PV cells with a well transversally organized tubular system, AC3 assembles near t-tubules. It should be noted that the nuclei of LA and LV are stained with AC3 in Figure 5A and B. It is unlikely that LV was captured under overexposed conditions compared to PV for the following reasons: (1) Neither the laser intensity of the microscope nor the gain during imaging is increased in these images compared to PV imaging in Figure 5C. (2) Microstructures in the transverse stripes were captured with similar resolution in LV and PV. 

### 3.4. Full Activation of L-Type Ca^2+^ Current by β-Adrenergic Receptor Agonist Requires Intracellular Ca^2+^ in the Supraventricular Area, Unlike in the Ventricles

**Figure 6:**
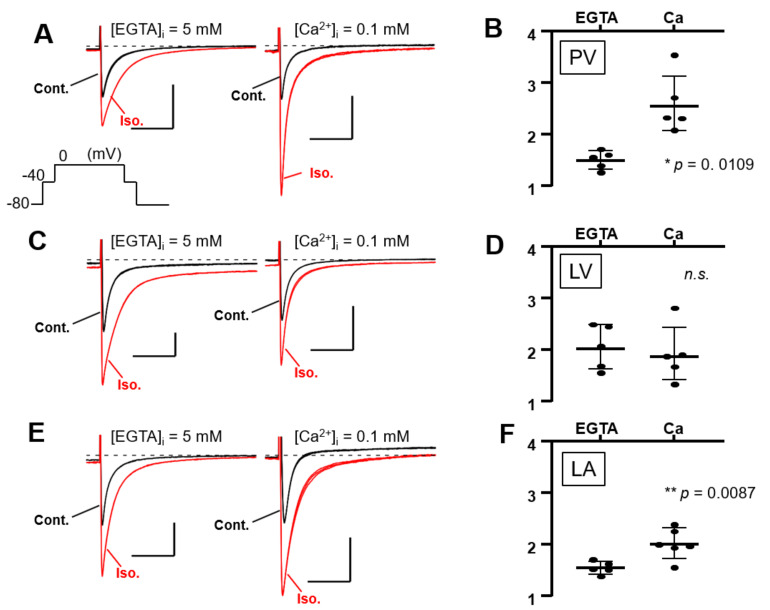
Ca^2+^-dependent effects of β-adrenergic stimulation on L-type Ca^2+^ currents in three types of cardiomyocytes. As shown in the inset of (**A**), the Ca^2+^ current is induced by a depolarizing pulse to 0 mV for 500 ms, followed by a depolarizing pulse to −40 mV for 80 ms to inactivate the Na^+^ current. The L-type Ca^2+^ current is amplified by 1 μM isoproterenol (Iso.), a β-adrenergic receptor-selective agonist. The strength of the β-adrenergic stimulation is evaluated by the rate of amplification of the current amplitudes. It was observed whether the absence or presence of intracellular Ca^2+^ changed the strength of the β-adrenergic reaction. In Figure 8A,C,E, three stable sweeps are superimposed under control conditions (Cont., black) or Iso. conditions (red). Inserted time and current scales indicate 50 ms and 5 pA/pF, respectively. Dashed lines indicate zero current level. Intracellular Ca^2+^ is chelated by 5 mM EGTA under the Ca^2+^-free condition, while the internal solution contains 0.1 mM Ca^2+^ in the other. (**A**) Representative data from pulmonary vein (PV) cardiomyocytes. A statistical summary of an increase in calcium current by Iso. for each condition is shown in (**B**). (**C**) Representative data from left ventricular (LV) cardiomyocytes and summarized in (**D**). (**E**) Representative data from left atrial (LA) cardiomyocytes and summarized in (**F**). *n.s.*, not significant.

**Figure 7:**
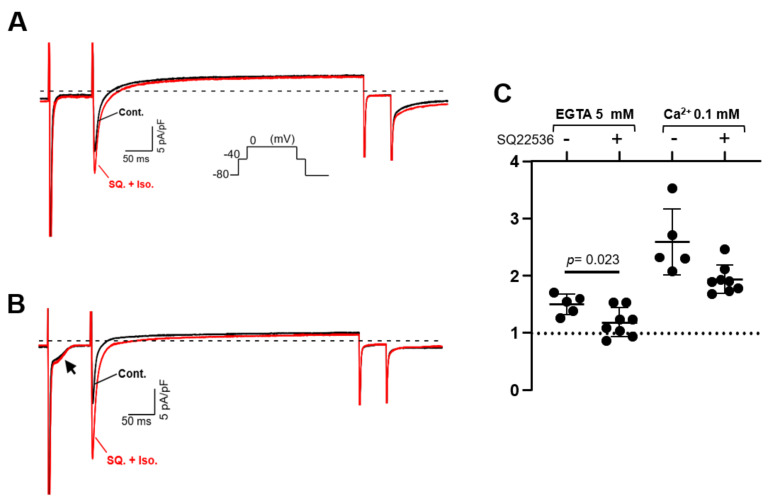
Effect of SQ22536 (SQ) on L-type Ca^2+^ current in pulmonary vein (PV) cardiomyocytes. The response of the Ca^2+^ current was recorded after 5 μM SQ application for approximately 2 min. The pulse protocol is inserted in (**A**). Intracellular Ca^2+^ is chelated by 5 mM EGTA in (**A**), while the internal solution contains 0.1 mM Ca^2+^ in (**B**). Dashed lines indicate zero current level. The rate of change in the current amplitudes for each condition is summarized in (**C**). Of note, under Ca^2+^-rich conditions, a Ca^2+^-dependent inward current indicated by an arrow in (**B**) is always recorded. This current is estimated to be of Na^+^-Ca^2+^ exchanger origin.

**Figure 8:**
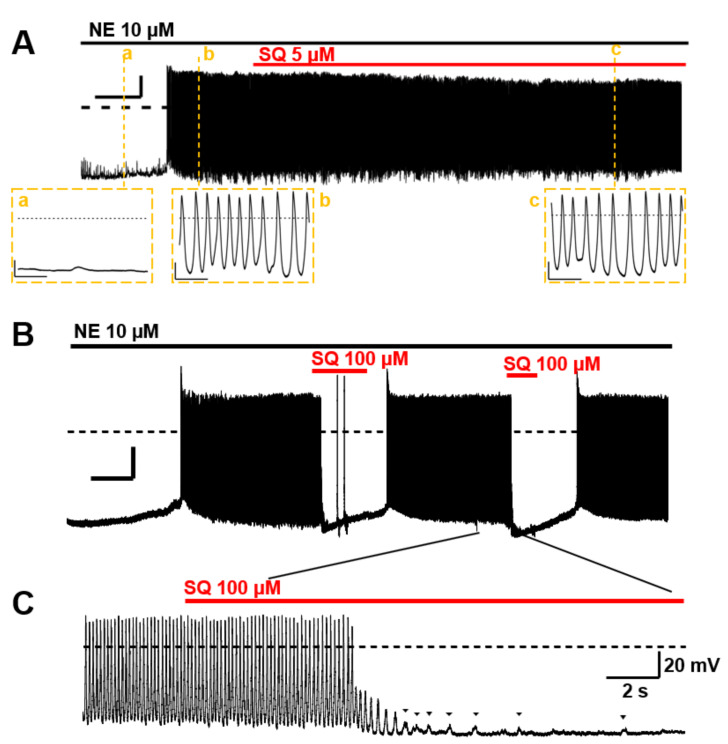
Effects of SQ22536 (SQ) on the norepinephrine (NE)-induced automaticity of pulmonary vein (PV). (**A**) An example of NE-induced spontaneous automaticity. NE 10 µM evoked a series of action potentials in isolated rat PV cardiomyocytes. The action potentials recorded at a, b, and c are displayed in an expanded time scale below. Inserted time and voltage scales indicate 50 s and 20 mV, respectively. The time scale in the expanded view indicates 500 ms. The induced automaticity was uninhibited by 5 µM SQ. (**B**) The induced automaticity was reversibly suppressed by 100 µM SQ. Inserted time and voltage scales indicate 50 s and 20 mV, respectively. The time course of the inhibition is expanded in (**C**). The sporadic depolarizations corresponding to Ca^2+^ release were gradually attenuated (arrowheads). Similar results were confirmed four times (*n* = 4, for each). The timing and duration of drug applications are indicated by horizontal bars. A dashed line indicates the baseline.

Isolated PV, LV, and LA cardiomyocytes were voltage-clamped by the patch-clamp technique. In L-type Ca^2+^ current recording, the extracellular solution was the K^+^-free Cs-Tyrode solution, and the K^+^-free internal solution was utilized. The Na^+^ current was inactivated by the depolarization step from a holding potential of −80 mV to −40 mV for 80 ms; subsequent depolarization to 0 mV activated the Ca^2+^ current. After confirming that the amplitude of the L-type Ca^2+^ current in our system hardly ran down within 10 min, all the recordings were completed within 10 min in this study. In Figure 6A,C,E, three stable sweeps are superimposed, one under control conditions (black) and one under isoproterenol-administrated conditions (red). Every Ca^2+^ current was enhanced by the β-adrenergic stimulation of 1 μM isoproterenol, and the intracellular Ca^2+^ concentration ([Ca^2+^]_i_) also affected the magnitude of the stimulant effect in PV myocytes. Under the [Ca^2+^]_i_-free condition with 5 mM EGTA, the β-adrenergic stimulation increased the Ca^2+^ currents amplitude of the PV cell by 1.50 ± 0.18 times. At [Ca^2+^]_i_ of 0.1 mM, the currents were further increased by 2.59 ± 0.58 times (Figure 6A,B). On the other hand, the currents of LV cells were magnified by 2.05 ± 0.43 and 1.91 ± 0.55 times under [Ca^2+^]_i_-free and -rich conditions, respectively (Figure 6C,D). There is no statistically significant difference between these values. In the LA cardiomyocytes, the Ca^2+^ currents were amplified by isoproterenol by 1.54 ± 0.12-fold in the chelated state of [Ca^2+^]_i_, while the currents were boosted to 1.97 ± 0.30-fold by the 0.1 mM [Ca^2+^]_i_ (Figure 6E,F). These data are summarized in Table 3 as well as Figure 6B,D,F. Of note, cells slowly repeated contractions and relaxations during the recording under [Ca^2+^]_i_-rich conditions. Table 3 reveals that the amplitude of the Ca^2+^ current is smaller under [Ca^2+^]_i_-rich conditions. This is presumably attributed to the characteristics of the L-type Ca^2+^ current, which is Ca^2+^-dependent inactivation [33]. We assessed the influence of AC3 activity on the property of PV cardiomyocytes by pharmacological experiments using an AC inhibitor. Because an AC3-selective reagent was not available, the nonspecific adenine-like inhibitor SQ22536 was employed for the pharmacological characterization. According to a previously published report, the IC_50_ of the inhibitor on AC3, AC5, and AC6 are 110, 3.5, and 5.8 µM, respectively, while AC9 is resistant to the drug [34]. In rat PV cardiomyocytes, the increase in the L-type Ca^2+^ current induced by isoproterenol is reduced by 5 µM of the inhibitor under the [Ca^2+^]_i_-free condition (Figure 7). The isoproterenol-induced augmentation of the Ca^2+^ current decreased from 1.50 ± 0.18 to 1.18 ± 0.23 (*p* = 0.023) in the absence of [Ca^2+^]_i_ (Figure 8A,B).

### 3.5. Suppression of Norepinephrine-Induced PV Automaticity by AC Inhibitor Treatment

The spontaneous automaticity evoked by 10 µM norepinephrine (NE) [5] could not be suppressed by 5 µM SQ22536 (*n* = 4, Figure 8A). However, the PV excitability was reversibly arrested by applying 100 µM (Figure 8B). The cessation of action potentials and gradual attenuation of transient depolarization was observed before it stopped completely (Figure 8C). These sporadic depolarizations originate from inward currents via NCX, which is evoked by transient Ca^2+^ elevation [5]. The NE-induced automaticity was always suppressed by 100 µM SQ22536 (*n* = 4). 

## 4. Discussion

### 4.1. Major Findings

The identity of the AC isoforms responsible for cAMP production in PV cardiomyocytes has not been previously elucidated. As a first step toward clarification of this critical issue, we employed RT-PCR, immunohistochemistry, and immunocytochemistry to determine the expression profiles in the rat heart and PVs. The gene expression profile of the AC isoforms by RT-PCR (Figure 1B) confirmed that all AC isoforms were expressed in the heart [35,36,37]; these results are consistent with previous studies that showed AC5 as the major isoform in cardiomyocytes. The most striking finding of the current study is that AC3 is specifically expressed in the supraventricular region, including PVs. In contrast, AC1, AC6, and AC8 were expressed throughout the heart and did not show reigion-specific expression patterns (Figure 2 and Figure 4A). AC2 protein was hardly detected in cardiomyocytes. In cell staining, AC3 was apparently localized along the t-tubule microdomain in PV cardiomyocytes with a well transversally organised tubular system (Figure 5). However, pulmonary vein cardiomyocytes showed a wide range of transverse tubular incidence and organization, going from their virtual absence, as described in atrial myocytes, to well transversally organized tubular systems, as in ventricular myocytes [11]. Therefore, the variability in the tubular system could also be associated with the cell-to-cell heterogeneity of AC3 localization in the PV myocardium compared with either the atrium or the ventricule. The t-tubule is an [Ca^2+^]_i_-concentrating apparatus. For example, during an action potential, the [Ca^2+^]_i_ below the t-tubule is estimated to exceed 200 μM [38,39]. Because AC3 is a Ca^2+^-stimulable isotype, there may be a connection between its location along the t-tubule and the reinforcement of the cAMP-dependent response by [Ca^2+^]_i_. In the patch-clamp experiments, the cAMP-dependent response of L-type Ca^2+^ currents in supraventricular cells, unlike ventricular cells, is enhanced when [Ca^2+^]_i_ is abundant (~100 μM, Figure 6). To evaluate the impact of ACs and their Ca^2+^ dependency on the arrhythmogenic property of PV, adenine-like AC inhibitor SQ22536 is administrated during the automaticity. An amount of 5 μM of this reagent suppressed the β-adrenegic stimulant effect on the Ca^2+^ current of PV under [Ca^2+^]_i_-free conditions, while the effects of the β-adrenergic response remain to a certain extent under [Ca^2+^]_i_-rich conditions (Figure 7). This drug fails to abolish the NE-induced automaticity at the same concentration (Figure 8A). By contrast, 100 µM of SQ22536 reproductively arrested the excitability (Figure 8B,C). Accordingly, these results suggest that Ca^2+^-stimulable AC3 plays the primary role in the NE-induced PV automaticity as a part of β-adrenergic signaling. To summarize the reasons: (1) Highly expressed Ca^2+^-stimulable AC3 localizes at the t-tubule at least in PV cardiomyocytes with a well transversally organised tubular system, where it is strongly influenced by [Ca^2+^]_i_. (2) The β-adrenergic effect evaluated by the Ca^2+^ current is enhanced by [Ca^2+^]_i_ in PV. Finally, (3) [Ca^2+^]_i_ is supposedly at a high level during the PV automaticity, and the inhibitory effect of SQ22536 on the automaticity is demonstrated at an adequate concentration of 100 µM against AC3, rather than AC5, AC6, or AC9. 

### 4.2. Potential Role of AC3 in the Rat Heart, among Other ACs

Among the nine membrane-bound AC isoforms, AC1, AC3, and AC8 are Ca^2+^-stimulable, while AC5 and AC6 are Ca^2+^-inhibitable [40]. Residual AC2, AC4, AC7, and AC9 are Ca^2+^-insensitive [41]. AC3 was first discovered in the olfactory epithelium [42] and was originally reported to be directly stimulated by Ca^2+^ and calmodulin [43]. In AC3 knockout mice (AC3^−/−^ mice), cAMP- and IP_3_-induced responses to odorants are completely abolished as measured by electro-olfactograms [44], urine and pheromones are not detected, and male sexual behavior is no longer observed [45]. However, as an inhibition of AC3 by the calmodulin-dependent protein kinase, which Ca^2+^ also activates, was recorded in vitro [46], the Ca^2+^-dependent activity of AC3 was controversial. Dr. Cooper’s team proved that AC3 is activated by submillimolar [Ca^2+^]_i_ in cellular experiments [47], whereas AC5 and AC6 were entirely inhibited by similar conditions [48,49]. In the current study, we estimated AC activities by the β-adrenergic response of L-type Ca^2+^ currents on a cell-by-cell basis. Consistent with LA and PV, where AC3 expression was regionally up-regulated, the β-adrenergic effect was enhanced by 0.1 mM [Ca^2+^]_i_ (Figure 6, Table 3). During sustained arrhythmias, such as the PV automaticity, [Ca^2+^]_i_ is kept high, and the activity of AC3 could be enhanced. In contrast, the activity of the major cardiac types, i.e., AC5 and 6, is declined. It should be considered that these AC types are apparently along the t-tubule (Figure 5 and [29]). Simulation studies predict that Ca^2+^ release from cardiac ryanodine receptors can increase [Ca^2+^]_i_ to >200 μM at the t-tubules [38,39]. As pharmacological charasteristics, SQ22536-sensitive isotypes are AC5 and 6 (IC_50_; 3.5 and 5.8 µM, respectively), moderately sensitive isotypes are AC1 and AC3 (IC_50_; 54 and 110 µM, respectively), and less sensitive are AC2, 4, and 7 (IC_50_; 210, 280, and 440 µM, respectively), while AC8 and AC9 are resistant to the drug [34]. Accordingly, the inhibitory effect of 100 µM SQ22536 in Figure 8B,C targets AC1 and AC3 rather than AC5, 6, and 9, indicating that Ca^2+^-stimulable AC may be involved in the catecholamine-dependent arrhythmogenic property of PV by producing cAMP under the high-[Ca^2+^]_i_ conditions. 

On the other hand, AC1 is believed to underlie normal automaticity in the sinoatrial node (SAN) [21]. AC1 is more highly expressed in the SAN than in the ventricles that lack automaticity. The basis of the coupled-clock theory of SAN beating is that a strong expression of AC1 in the localized region produces the unique calcium dynamics via cAMP-dependent phosphorylation of ryanodine receptors and phospholamban [50]. In our experiments, AC1 is not more highly expressed in the pulmonary veins than in the ventricles, so we propose that the pulmonary venous automaticity is derived from AC3, not AC1, as an analogy to the coupled-clock theory. As for cardiac majors, AC5 and AC6 are very closely related isotypes and are believed to be dominantly expressed in the heart. In rats, AC5 expression becomes more dominant with advancing age [51], and AC5^−/−^ mice have been found to have impaired responses to sympathetic stimulation as well as parasympathetic responses such as baroreceptor reflexes [52]. In addition, AC6^−/−^ mice dramatically reduce not only AC6 but also AC5 protein expression. Therefore, the phenotype of AC6^−/−^ mice is stronger than that of AC5^−/−^ mice, and the positive inotropic effects induced by dobutamine is greatly impaired. This is because Ca^2+^-related proteins such as phospholamban are less likely to be phosphorylated by cAMP-dependent kinase [53]. In recent years, AC9 has been extensively studied [54,55,56]. Although cAMP production by AC9 is less than 3% of the total cardiac cAMP production, AC9^−/−^ mice show impaired cardiac diastolic capacity and reduced heart rate [24]. It is thought that AC9 binds to KCNQ1 channels via A-kinase anchor protein and promotes the repolarization phase of the action potential during sympathetic nerve stimulation, thereby increasing heart rate [57]. 

As described above, while AC1, AC5, AC6, and AC9 have been reported as the main physiological cAMP producers in the heart, AC3 is distinguished by its pathophysiological function.

### 4.3. Interpretation of Region-Dependent AC3 Expression

Surprisingly, immunohistochemistry with anti-AC3 revealed four heart regions. The AC3-negative ventricular region was separated from the AC3-positive supraventricular area. The PV was heterogeneously stained, whereas the atrium was uniformly stained. The high AC3 expression on the inner side of the vein was isolated from the unstained zone in the proximal and peripheral portions of the vessel. The SAN was an unstained island in the supraventricular area. To our knowledge, this is the first case in which simple staining has discerned four heart regions. Indeed, these regions are known to undergo different developmental processes. For example, several transcription factors appear in heart region-specific manners during early developmental stages [58]. *Irx4* [59] and *Hrt2* [60] are strongly stained by in situ hybridization in the ventricular precursor region during embryonic day 9.5–10.5, whereas *Hrt1* expression is restricted to the atrial precursor region. *Pitx2c* is required for cardiomyocyte proliferation in the PV after developing the atrial and ventricular precursors during embryonic days 11.5–12.5 [61]. *Shox2* directs the expression of *Isl1* in the SAN, which establishes the pacemaker activity by suppressing needless genes before the embryonic heart starts to beat regularly [62,63]. These transcription factors are core molecules that organize the region-dependent expression of functional proteins such as hormones, enzymes, and ion channels. AC3 can thus be considered as one region-specific enzyme. 

### 4.4. Limitations

The main limitation of our study is the absence of direct experiments that specifically target AC3, such as selective inhibitors of AC3, genetic recombination experiments with AC3, or knockout mice for AC3. In addition, we have not been able to establish a method to measure cAMP at the cellular level, so we can only indirectly analogize the function of AC by evaluating the amplitude of the Ca^2+^ current. Thus, even though it is certain that AC3 is strongly expressed in the supraventricular region of the heart, direct experiments on the function of AC3 in the heart are still insufficient. We hope that future advances in materials will reproduce and strengthen our conclusion that AC3 is involved in PV arrhythmias.

## 5. Conclusions

This study identified specific AC3 expression in the rat supraventricular area, including arrhythmogenic PV cardiomyocytes. In the t-tubule membrane structure of PV cells, Ca^2+^-stimulable AC3 may play an arrhythmogenic role in norepinephrine-induced automaticity under high-[Ca^2+^]_i_ conditions, rather than other Ca^2+^-inhibitable and -insensitive ACs. Accordingly, SQ22536 suppressed the automaticity potently and reversibly at the concentration appropriately against AC3. These findings suggest that the blockade of AC3 can suppress the ectopic excitability of PVs. Our data may provide valuable information on how ectopic excitability occurs in PVs and may contribute to the development of drugs that can pharmacologically suppress the Ca^2+^-dependent arrhythmogenic property of PVs because AC3-selective reagents are currently unavailable.

## Figures and Tables

**Table 1:** List of PCR primers for rat AC1–8 and GAPDH. Specificities were checked in the database NCBI-BLAST (https://blast.ncbi.nlm.nih.gov/Blast.cgi (accessed on 1 January 2020), and accuracies were checked by the singular melting peaks using LightCycler. RT-PCR products on the Tris-borate-EDTA gel. The rat GAPDH primer is routinely used in our experiments [23].

	Forward Sequences	Reverse Sequences
**rAdcy1**	5’-TATCCTGCTGTTCTCATGCACG-3’	5’-TTGGACATGAGGAAGTGCTGTG-3’
**rAdcy2**	5’-ATCATCAGGCATCATCGCCAAC-3’	5’-ATTCACTCTGTCTGCCCAGAAC-3’
**rAdcy3**	5’-ATGGTGAAGCTGACACTCATGC-3’	5’-AAATGGCGTGCAACATGCTC-3’
**rAdcy4**	5’-TGCCAAGTTCTTCCAGGTCATC-3’	5’-TGGGAGTGCAGAAATAGGGAAC-3’
**rAdy5**	5’-ATTGACAGACTGCGATCCGAAC-3’	5’-ATGACATGCGTTCACCTGGTTC-3’
**rAdcy6**	5’-ATGGCAGTTTGATGTCTGGTCC-3’	5’-ATCGGACGGTGTTAAGTTCAGC-3’
**rAdcy7**	5’-ATGAACAGCCACACCCTTGCTC-3’	5’-GCTCCTTCTTAAACTTCTTCT-3’
**rAdcy8**	5’-AGAATACTCTGGCTGCCCTAAC-3’	5’-AGCCTCGAAGAAGGAAGCAAAC-3’
**rGapdh**	5’-ACCACAGTCCATGCCATCAC-3’	5’-TCCACCACCCTGTTGCTGTA-3’

**Table 2:** Results of simplified-absolute PCR quantification. Statistical *p*-values for each isotype are given below the table. The number of experiments is provided in parentheses.

	×10^9^	LA		LV		PV	
*corresponding abundance*	AC1 *	53,200.08 ± 78,097.71	(7)	340,281.06 ± 402,755.99	(5)	876,466.31 ±1,272,990.19	(6)
	AC2 ^#^	1.95 ± 2.79	(6)	3.38 ± 3.79	(3)	3.52 ± 4.59	(5)
	AC3 ^$^	80,931.17 ±126,819.78	(6)	46,348.88 ± 57,065.14	(5)	150,449.16 ± 301,138.14	(6)
	AC4 ^†^	20.37 ±28.00	(6)	9.43 ± 8.09	(4)	12.32 ± 12.63	(5)
	AC5 ^&^	1,451,129.43 ±3,541,141.54	(8)	408,442.32 ± 785,280.03	(5)	536,885.37 ± 661,839.27	(5)
	AC6	549.27 ±730.34	(8)	417.79 ± 681.51	(5)	810.37 ± 1038.31	(5)
	AC7	4051.16 ± 6371.10	(7)	393.87 ± 725.97	(5)	29,443.41 ± 39,503.56	(5)
	AC8	130.21 ± 168.44	(5)	35.74 ± 67.52	(5)	167.89 ± 205.04	(5)

* *p* < 0.0001, vs. AC2; *p* < 0.0001, vs. AC4; *p* = 0.0029, vs. AC6; *p* = 0.0026, vs. AC7; *p* < 0.0001, vs. AC8. ^#^ *p* = 0.0002, vs. AC3; *p* < 0.0001, vs. AC5. ^$^ *p* = 0.0011, vs. AC4; *p* = 0.0048, vs. AC8. ^†^ *p* < 0.0001, vs. AC5. ^&^ *p* = 0.0014, vs. AC6; *p* = 0.0012, vs. AC7; *p* < 0.0001, vs. AC8.

**Table 3:** Comparison of L-type Ca^2+^ current amplitudes under different conditions. The current amplitudes are an average of three stably recorded sweeps. *Baseline* and *Isoproterenol* conditions indicate the current amplitudes in the absence and presence of 1 μM isoproterenol, respectively. The amplitude in the presence of isoproterenol is divided by the baseline to calculate the effect. Statistical *p*-values for the differences between the values are given below the table. PV, pulmonary vein; LA, left ventricle; LA, left atrium; Cm, membrane cell capacitance.

*Cell Type*	*[Ca^2+^]_i_*	*Baseline (pA/pF)*	*Isopreterenol (pA/pF)*	*Effect (-Fold)*	*Cm (pF)*	*n* =
*PV*	[EGTA]_i_ = 5 mM	9.45 ± 4.18	14.27 ± 6.89	* 1.50 ± 0.18	194.80 ± 76.97	5
*PV*	[Ca^2+^]_i_ = 0.1 mM	7.07 ± 1.92	17.67 ± 3.24	* 2.59 ± 0.58	230.28 ± 40.64	5
*LV*	[EGTA]_i_ = 5 mM	11.0 ± 3.14	21.66 ± 3.97	2.05 ± 0.43	223.07 ± 51.45	5
*LV*	[Ca^2+^]_i_ = 0.1 mM	6.86 ± 2.76	12.62 ± 4.96	1.91 ± 0.55	246.05 ± 40.62	5
*LA*	[EGTA]_i_ = 5 mM	14.1 ± 2.54	21.55 ± 3.12	^#^ 1.54 ± 0.12	117.13 ± 41.96	5
*LA*	[Ca^2+^]_i_ = 0.1 mM	9.47 ± 4.49	17.74 ± 5.47	^#^ 1.97 ± 0.30	141.76 ± 38.50	6

* *p* = 0.0109; ^#^ *p* = 0.0087.

## Data Availability

The data in this study are available on a request basis.
